# Links between chemsex and reduced mental health among Norwegian MSM and other men: results from a cross-sectional clinic survey

**DOI:** 10.1186/s12889-020-09916-7

**Published:** 2020-11-25

**Authors:** Rigmor C. Berg, Eirik Amundsen, Åse Haugstvedt

**Affiliations:** 1 Norwegian Institute of Public Health, Postboks 222 Skøyen, 0213 Oslo, Norway; 2 University of Tromsø, Hansine Hansens veg 18, N-9019 Tromso, Norway; 3 Oslo University Hospital, Nasjonal kompetansetjeneste for seksuelt overførbare infeksjoner, Postboks 4763, 0506 Oslo, Norway

**Keywords:** Chemsex, Men who have sex with men, Mental health, Substances, Norway

## Abstract

**Background:**

The use of specific drugs to facilitate, enhance or prolong sexual sessions is referred to as ‘chemsex’. The popularity of the behavior seems to be growing, but there is a paucity of information on the mental health aspects associated with chemsex and no data on chemsex from Nordic countries. We investigated the link between chemsex and mental health among men who have sex with men (MSM) and other men in Norway.

**Methods:**

We recruited participants from a walk-in sexually transmitted infections (STI) clinic. Participants completed a piloted, anonymous self-administered survey. It consisted of questions about men’s sociodemographic characteristics, mental health, sexual behaviors, substance use, and chemsex. The outcome we investigated was reduced mental health, measured with the validated Hopkins Symptom Check List. We obtained descriptive statistics and performed univariate and multivariate logistic regression analyses.

**Results:**

1013 (96%) of the surveys were complete and could be analysed. The mean age of the sample was 33, 51% were MSM, and 21.7% had reduced mental health. More MSM than other men engaged in chemsex in the past year (17% vs 12%). The most frequently reported chemsex drugs were cocaine and gamma hydroxybutyrate/gamma butyrolactone (GHB/GBL). Men engaged in chemsex primarily to enhance sexual pleasure and excitement, and about half reported almost never or never using condoms for chemsex. In univariate analyses, significant predictors of reduced mental health was chemsex (Odds Ratio [OR] = 1.82), being unemployed (OR = 3.54), and having sex with only women (OR = 0.58). In multivariate analyses, two variables remained significantly associated with reduced mental health: chemsex (adjusted OR = 2.18, 95%CI = 1.25–3.78) and being unemployed (adjusted OR = 4.10, 95%CI = 2.13–7.87).

**Conclusions:**

In our sample of men from an STI clinic in Norway, about 14% self-reported engaging in chemsex in the past year and about a fifth of the men had reduced mental health. Men who engaged in chemsex, which more MSM engaged in than other men, had two times greater odds of reduced mental health. These findings suggest that mental health assistance should be among the interventions offered to men engaging in chemsex.

**Supplementary Information:**

The online version contains supplementary material available at 10.1186/s12889-020-09916-7.

## Background

The use of recreational substances for sex is not a new phenomenon. Sex while intoxicated or high occurs in both men and women and across sexual orientations, although there is mounting evidence that such drug use is more common among sexual minorities [1, 2]. These days, according to a global drug survey, the most commonly used drugs with sex are alcohol, cannabis, and MDMA (methylenedioxymethamphetamine) [1]. Recently, the use of specific recreational drugs to facilitate, enhance or prolong sexual sessions is increasingly referred to as ‘chemsex’ [3, 4]. The drugs most commonly used are methamphetamines, gamma hydroxybutyrate/gamma butyrolactone (GHB/GBL), mephedrone, cocaine, and ketamine [4, 5]. Although there is limited systematic data available, existing scholarship shows that while such substances are used with sex by both men having sex with men (MSM) and men having sex with women, chemsex is considerably more common among MSM than other men. Lawn and colleagues [1] found that more MSM than non-MSM used cocaine, GHB/GBL, ketamine, mephedrone, and methamphetamine with sex. Use in the past 12 months among MSM varied from 4.2% (mephedrone) to 14.1% (cocaine) and among non-MSM from 0.7% (GHB/GBL) to 10.1% (cocaine). Also a review of chemsex shows that prevalence estimates of chemsex among MSM vary greatly across various samples and measurement and recruitment methods used, ranging from 3 to 29% [4]. Longitudinal research demonstrates mixed evidence on prevalence changes over time in the use of chemsex drugs among MSM [6, 7].

Chemsex has been enabled by the last decade’s geospatial networking applications such as Grindr and other online sites to meet sexual partners [3], but there are also underlying physical, emotional, and social drivers for engaging in chemsex. These appear to be complex, but there are indications that those who engage in chemsex are seeking a powerful sexual experience. The typical chemsex drugs are stimulants that provide feelings of euphoria, heightened sexual arousal, and increased stamina which facilitate long sexual sessions with multiple partners [4, 8, 9]. Qualitative interviews suggest that reasons for engagement in chemsex include not just the enhanced sexual and physical sensations [10], but also cognitive disengagement, intensified perceptions of intimacy, and overcoming social inhibitions as well as stigma, marginalisation, and minority stress [10, 11]. Pollard and colleagues [11] highlight how chemsex, as an individual and social behavior, exists within syndemics of marginalisation and health inequality. They point to research such as those by Berg and colleagues [12, 13], showing that an insidious consequence of heteronormativity and minority stress is the internalisation of that stigma by sexual minorities, manifest as e.g. internalised homonegativity, which in turn is a predisposing factor in several aspects of ill health, such as loneliness. Research has revealed that syndemics of marginalisation of gay, bisexual, and other MSM are social determinants of health challenges like HIV-risk, mental health burdens, illicit drug use, and sexual risk taking [2, 11]. In this conceptualisation, chemsex and its social context are mutually constituted [11].

While the use of ‘chem’-drugs, regardless of who consumes them, can have serious implications for those taking them because of the direct negative effects of the drugs themselves [14, 15], there are also inter-connected risk behaviors associated with combining chem-drugs and sex. Two recent literature reviews [4, 16] concluded that MSM who engage in chemsex are more likely than men who do not engage in chemsex to engage in condomless anal intercourse, perform esoteric sex acts such as fisting and group sex, and to be diagnosed with sexually transmitted infections (STIs) and HIV. The rise of chemsex as a public health issue may be due to its associated sexual risk-taking, but there are also issues around sexual consent, difficulties in negotiating sex [3], destruction of social and romantic relationships, and chemsex as a maladaptive coping strategy for painful emotions [11].

While there appears to be generous research on the association between chemsex and physical health, such as STIs, the authors of the literature reviews of chemsex related behaviors [4, 16] identified only a handful of studies on the psychosocial impacts of chemsex. Overall, these studies from the UK, USA and Australia suggested that chemsex drug use negatively affected men’s lives, including employment, social networks, and daily functioning. For example, two studies from sexual health clinics in London identified that 15 and 42% of the MSM respondents, respectively, perceived chemsex to have an adverse impact on their mental health [17, 18]. A US-based study, with focus groups of 15 MSM, recorded experiences of paranoia, short-term depression, and psychosis following chemsex engagement [19]. However, none of these studies examined the statistical association between chemsex and mental health. This knowledge gap is recently beginning to be addressed, notably by three UK-based studies. First, both a national probability sample among sexually active HIV-positive MSM and a sample of HIV-negative MSM, recruited from sexual health clinics, found that chemsex was associated with self-reported ever diagnosis of depression or anxiety [20] and symptoms of depression and anxiety [7]. However, the associations were not significant once other factors were controlled for. Similarly, while an online survey found that sexualised drug use – which included use of substances such as alcohol and erectile dysfunction drugs just before or during sex – was correlated with lower satisfaction with life, the multivariate analyses showed no significant differences in psychological distress, internalised homonegativity, loneliness, or satisfaction with life between MSM engaging in sexualised drug use and MSM engaging in chemsex [9]. In another UK-based study, chemsex drug use (which does not necessarily equate to engaging in chemsex) was associated with symptoms of depression [21].

Although there appears to exist few studies on chemsex from Nordic countries [4, 16], we identified a recent cross-sectional study from 13 European cities, including the capital of Sweden, which examined the prevalence and predictors of drug use during the last sexual encounter [22]. It found that 3.4% of the 4266 MSM respondents reported the use of chemsex drugs (GHB/GBL, ketamine, mephedrone, crystal methamphetamine) during last anal sex with a male partner. Prevalence across the 13 cities ranged from 0 to 13.9%, with only one man residing in Stockholm reporting such use. Chemsex was strongly associated with recent STI diagnosis, younger age, history of injecting drug use, and sexual encounters with more than one partner. It bears mention that one older Internet-based study among MSM from Norway found that 10% reported being under the influence of selected drugs during sex in the past year, and the behavior was associated with being diagnosed with HIV. However, the drugs were not specifically chemsex drugs, rather included marihuana, prescription drugs, ecstasy, LSD, GHB, cocaine, heroin, amphetamines, and methamphetamines [23].

It is clear that the use of chemsex drugs and chemsex vary greatly across cities and sub-groups, partially because laws and social attitudes to drugs are likely to influence use and that the concept of chemsex is socially constructed. To date, there are limited data on chemsex among non-MSM, men residing in Nordic countries, and the link between chemsex and mental health. Thus, given growing international evidence of the popularity of chemsex, the potential risks associated with the use, the limited data on the mental health aspects, and the paucity of information on this issue in Nordic countries, we investigated the link between chemsex and mental health among MSM and other men attending a sexual health clinic in Norway.

## Methods

The methods are reported according to the STROBE guidelines for observational studies [24]. Study procedures were reviewed and approved by the Regional Committee for Medical and Health Research Ethics, region south east Norway. The Data Protection Impact Assessment was approved through the Oslo University Hospital.

### Study population

We recruited participants from a walk-in STI clinic in Norway. Eligibility criteria were: (1) being over 16 years (age of consent); (2) birth-assigned male sex; (3) had sex in the last year; (4) being able to read Norwegian or English.

### Recruitment and procedures

Recruitment occurred between July and October 2016 at the largest STI clinic in Norway (Olafiaklinikken), in Oslo. It is a low-threshold – most services are free of charge – drop-in clinic for the diagnosis and treatment of STIs. The receptionists informed male patients about the study. Eligible men who expressed interest were asked to complete the paper-and-pencil survey and subsequently place it in a locked box located in the waiting room. The survey was available in English and Norwegian. It took 5–10 min to complete. All data collected were anonymous.

### Measures

We piloted the first version of the survey, which was specifically developed for this study, with 20 patients (not included in the final dataset). They provided detailed feedback on the contents, functionality, and survey layout. After revisions, the final survey comprised 48 questions that fit on two sheets of paper (supplementary file 1). We used the validated Hopkins Symptom Check List (HSCL-10) to assess mental health, the dependent variable. It has high reliability (Cronbach’s alpha = 0.88), high validity (89% sensitivity, 98% specificity), behaves in the same way in different socio-demographic groups (e.g. age, gender, level of education, employment), and shows high correlation with similar instruments (0.91–0.97), which indicates that the same characteristics are measured [25]. Reliability in the current sample was 0.91. HSCL-10 consists of 10 symptoms of distress (depression and anxiety) that people may have experienced in the last 2 weeks. Respondents were asked to assess each item using a 4-point Likert scale ranging from 1 = “Not at all”, 2 = “A little”, 3 = “Quite a bit”, to 4 = “Extremely”. The average score is calculated by dividing the total score by the number of items, with a theoretical range of 1–4, and a reduced mental health cutoff ≥1.85 [25]. Another five single items probed further details of mental health, including suicidal thoughts.

With respect to the other variables, we asked about sociodemographic characteristics, sexual orientation and behaviors, STI/HIV history and testing, and substance use. These were adapted from research on similar topics [26]. We had 15 questions about chemsex, with the wording for chemsex, the independent variable of interest, being “In the last 12 months, how often have you taken drugs immediately preceding and/or during the sexual session (i.e., engaged in ‘chemsex’)? Drugs include methamphetamine, GHB/GBL, mephedrone, ketamine, cocaine”. We used the term chemsex in both Norwegian and English. We specified the five drugs in accordance with existing evidence [5].

### Statistical analyses

SPSS 23.0 statistical software was used to perform analyses. We used descriptive statistics to examine sample characteristics and proportions with reduced mental health. To examine differences between MSM and non-MSM we used Chi-square tests. We conducted first univariate analyses and next a multivariate analysis using logistic regression with reduced mental health as outcome variable. We used the enter method with intercept, including eight explanatory variables that met collinearity requirements. Analyses were two-tailed with significance set at the 5% level.

## Results

Of the 1050 surveys handed out, 1013 (96%) were complete and could be analysed. As shown in Table 1, the mean age of the sample was 33, three quarters had a university degree and worked full- or part-time, and 60% described themselves as single. Half of the sample self-described as gay or bisexual, and a little more than half of the sample (50.7%) self-reported having sex with men in the past year (MSM). For this study, MSM are defined as men who self-reported having sex with men in the past year. Nine of ten respondents had lived in Norway for more than ten years. Not shown is that 44 (4.3%) self-reported living with HIV (all except one man was MSM). Further details of the sample are available in a separate Norwegian report [27].

**Table 1:** Sociodemographic characteristics of the sample and subgroups

	Full sample (*N* = 1013) %	Chemsex (*n* = 144) %	No chemsex (*n* = 857) %
Age (Mean [SD], range)	33.2 (9.2), 18–79	33.0 (8.0), 20–55	33.2 (9.4), 18–79
Current residency			
Oslo	936 (92.8)	138 (95.8)	784 (91.5)
Elsewhere	73 (7.3)	5 (3.5)	69 (8.1)
Education			
Primary school	26 (2.6)	4 (2.8)	20 (2.3)
High school/Secondary school	229 (22.6)	39 (27.1)	188 (21.9)
College/University degree	477 (47.1)	68 (47.2)	401 (46.8)
Master or Ph.D	280 (27.6)	32 (22.2)	246 (28.7)
Employment			
Work full- or part time	758 (74.7)	107 (74.3)	642 (74.9)
Student	179 (17.6)	24 (16.7)	152 (17.7)
Unemployed	50 (4.9)	12 (8.3)	37 (4.3)
Retired/On disability/Other	28 (2.8)	1 (0.7)	26 (3.0)
Relationship status			
In relationship with man	186 (18.4)	35 (24.3)	149 (17.4)
In relationship with woman	213 (21.0)	21 (14.6)	185 (21.6)
Single	605 (59.8)	88 (61.1)	512 (59.7)
Other	8 (0.8)	0	8 (0.9)
Sexual orientation			
Gay/homosexual	438 (43.4)	74 (51.4)	361 (42.1)
Bisexual	71 (7.0)	12 (8.3)	58 (6.8)
Heterosexual	496 (49.2)	58 (40.3)	430 (50.2)
Other	4 (0.4)	0	4 (0.5)
Years lived in Norway			
< 5 years	59 (6)	9 (6)	50 (6)
5–10 years	65 (6)	14 (19)	51 (6)
> 10 years	882 (87)	121 (84)	761 (89)

With respect to mental health, our outcome of interest, we found that 21.7% of the sample had reduced mental health (score ≥ 1.85 on symptoms of depression and anxiety). Moreover, 9% were currently receiving therapy from a psychologist or psychiatrist, 2.7% had attention deficit hyperactivity disorder, and in the past year 3.3% had seriously considered suicide, 0.5% had attempted suicide, and 0.4% had been admitted to a psychiatric ward. With regard to these five variables, there was no statistically significant difference between those who engaged in chemsex and those who did not (*p* = 0.94, 0.15, 0.13, 0.99, 0.99, respectively).

### Description of chemsex experiences

All but 12 men (*n* = 1001) answered the questions about chemsex, of which 144 (14.4%) reported having engaged in chemsex in the past year. Table 2 shows the characteristics of chemsex use among these men. Three quarters of men reporting chemsex in the past year had engaged in chemsex two or more times, and the most frequently reported chemsex drugs were cocaine and GHB/GBL. They engaged in chemsex primarily to enhance sexual pleasure (57%), excitement (47%), and ability (24%). The location for the activity was largely in a private home (73%) and about half reported almost never or never using condoms. About one in ten had a few times experienced physical problems or psychological problems related to chemsex, and two in ten had engaged in activities during chemsex that they regretted. 13% stated they had ever wanted to quit chemsex. Slightly more MSM than other men engaged in chemsex (17% vs 12%). Significantly more MSM than non-MSM used GHB/GBL, engaged in chemsex to enhance sexual pleasure and excitement, found partners via the Internet or App or sauna, and had chemsex in private homes, sauna or sex clubs. Not shown is that more MSM also had a higher number of sex partners and more STIs. One man reported having been hospitalised for medical issues caused by chemsex, six reported ‘slamming’ (injecting drugs) and one sharing of needles in relation to chemsex.

**Table 2:** Characteristics of chemsex, among respondents ever having engaged in chemsex (*n* = 144)

Variable	Full sample n (%)	MSM n (%)	non-MSM n (%)	Test for stat. Diff
Frequency of chemsex				*p* = 0.35
Once	36 (25)	19 (22)	17 (30)	
Two or more times	108 (75)	68 (78)	40 (70)	
Drugs used for chemsex ^1^				
Cocaine	90 (63)	45 (52)	45 (79)	*p* = 0.22
GHB/GBL	41 (29)	37 (43)	4 (7)	*p* = 0.001*
Methamphetamine	24 (17)	20 (23)	4 (7)	*p* = 0.11
Ketamine	16 (11)	9 (10)	7 (12)	*p* = 0.28
Mephedrone	9 (6)	8 (9)	1 (2)	*p* = 0.05
Reasons for engaging in chemsex ^1^				
Increased sexual pleasure	82 (57)	57 (66)	25 (44)	*p* = 0.006*
Increased sexual excitement	67 (47)	46 (53)	21 (37)	*p* = 0.017*
Increased sexual performance	34 (24)	22 (25)	12 (21)	*p* = 0.78
Low self-esteem	8 (6)	5 (6)	3 (5)	*p* = 0.87
Pressure from partner	5 (3)	4 (5)	1 (2)	*p =* 0.35
Other	28 (19)	9 (10)	19 (33)	*p =* 0.001*
Means/location of finding chemsex partner ^1^				
Internet/App	52 (36)	49 (56)	3 (5)	*p =* 0.001*
Sauna	6 (4)	6 (7)	0	*p* = 0.042*
Sex-club	7 (5)	5 (6)	2 (4)	*p* = 0.52
Cruising place	4 (3)	2 (2)	2 (4)	*p* = 0.69
Other	68 (47)	26 (30)	32 (56)	*p =* 0.22
Location/place for chemsex ^1^				
Private home	105 (73)	68 (78)	37 (65)	*p* = 0.043*
Sexparty in private home	29 (20)	28 (32)	1 (2)	*p =* 0.001*
Hotel	30 (21)	21 (24)	9 (16)	*p* = 0.38
Sauna	9 (6)	9 (10)	0	*p* = 0.011*
Sexclub	8 (6)	8 (9)	0	*p =* 0.017*
Cruising place	3 (2)	2 (2)	1 (2)	*p* = 0.80
Other	7 (5)	1 (1)	6 (11)	*p* = 0.012*
Used condoms during chemsex				*p* = 0.06
Almost never/never	67 (47)	37 (43)	30 (53)	
A few times	16 (11)	11 (13)	5 (9)	
Almost always/always	38 (26)	31 (36)	7 (12)	
Had physical problems because of chemsex				*p* = 0.76
Almost never/never	103 (72)	67 (77)	36 (63)	
A few times	13 (9)	9 (10)	4 (7)	
Almost always/always	5 (3)	4 (5)	1 (2)	
Had psychological problems because of chemsex				*p* = 0.81
Almost never/never	100 (87)	66 (76)	34 (60)	
A few times	15 (10)	11 (13)	4 (7)	
Almost always/always	5 (3)	3 (3)	1 (2)	
Engaged in sexual activities during chemsex that you later regretted				*p* = 0.57
Yes	27 (19)	19 (22)	8 (14)	
No	70 (49)	45 (52)	25 (44)	
Unsure/Can’t remember	22 (15)	14 (16)	8 (14)	
Wish to stop engaging in chemsex				*p* = 0.97
Yes	18 (13)	13 (15)	5 (9)	
No	53 (37)	37 (43)	16 (28)	
Unsure	49 (34)	28 (32)	21 (37)	

Legend: 1 = respondents could check more than one answer. * = *p* < 0.05

### Association between reduced mental health and selected variables

As seen in Table 3, in univariate analyses, significant predictors of reduced mental health were chemsex (odds ratio [OR] = 1.82), being unemployed (OR = 3.54), and having sex with only women (OR = 0.58). In the multivariate analysis, two variables remained significantly associated with reduced mental health: chemsex (adjusted OR = 2.18, 95%CI = 1.25–3.78) and being unemployed (adjusted OR = 4.10, 95%CI = 2.13–7.87).

**Table 3:** Bivariate and multivariable logistic regression analysis of influence of chemsex on reduced mental health

Variable	OR (95% CI)	β (SE)	aOR (95% CI)
Chemsex	1.82 (1.19–2.76)	0.78 (0.28)	2.18 (1.26–3.79) **
Age	0.99 (0.98–1.01)	−0.01 (0.01)	0.99 (0.97–1.02)
HIV-positive	0.70 (0.30–1.61)	−0.65 (0.55)	0.52 (0.18–1.54)
Unemployed	3.54 (2.09–6.00)	1.41 (0.33)	4.10 (2.14–7.88) **
Years lived in Norway	0.82 (0.46–1.48)	−0.44 (0.37)	0.64 (0.31–1.32)
Steady relationship	1.16 (0.84–1.60)	−0.05 (0.22)	0.95 (0.62–1.47)
Sex with only women	0.58 (0.35–0.96)	−0.06 (0.35)	0.94 (0.48–1.87)
Used Internet to find sexual partners	0.94 (0.64–1.38)	−0.02 (0.25)	0.99 (0.61–1.60)

Legend: ** = *p* < 0.001

## Discussion

This is the first study from a Nordic country to demonstrate that chemsex occurs among both MSM and non-MSM and is associated with reduced mental health. While several studies show that use of drugs is more common among MSM than non-MSM [6, 28, 29], we are only aware of one other study with both MSM and non-MSM that has examined the use of specific recreational drugs used shortly before the sexual session [1]. Similar to the study by Lawn and colleagues [1], we found that chemsex was somewhat more common among MSM (17% vs 12%), they were more likely to prefer GHB/GBL and used the drugs to enhance the sexual experience. MSM differed from non-MSM also in means of finding chemsex partners and location of having chemsex, relying more on Apps and private homes. Otherwise, there were surprisingly few differences and more research is encouraged to continue to explore potential differences in the phenomenon of chemsex between the two groups.

In our sample of 1013 men from a walk-in STI clinic in Norway, about a fifth of the men had reduced mental health. The rate was similar between MSM and non-MSM (22.6% vs 19.0%). Relative to three other clinic-based studies on chemsex, this is higher than the rate of clinically significant depressive or anxiety symptoms [7], similar to the rate of psychological distress [9], but lower than the rate of ever being diagnosed with depression or anxiety [20]. The modest differences are likely partly explained by differences in measurement methods used. Related, in the past year, 3.3% had seriously considered suicide and 0.5% had attempted suicide. While suicidal thoughts and attempts signal reduced mental health, these outcomes were not statistically associated with chemsex (but reduced mental health was), possibly due to the low numbers reporting such problems and this being associated with other factors. Without doubt, from a public health standpoint, these data on mental health are worrying, as mental health problems is one of the leading causes for loss of productivity and lower quality of life. Some research shows that MSM experience higher rates of mental health problems compared with other men [30], and that people living with HIV are at increased risk of mental health problems [31]. Our analysis from an STI clinic found that neither MSM nor HIV-positive men were more likely than other men to report symptoms of depression and anxiety. This is similar to recent UK-based research [9, 18]. Yet, we note that meta-analyses of population-based studies have concluded that the lifetime prevalence of suicide attempts in gay/bisexual males is two to four times that of comparable heterosexual males [32].

We found that reduced mental health was independently associated with engaging in chemsex and being unemployed. To the best of our knowledge, and as indicated by reviews about use of drugs for sexual purposes [4, 16], our study is one of few to evaluate the independent association between chemsex and mental health. This association was robust when potentially confounding factors were controlled for. Our finding is confirmatory of other qualitative and quantitative research on gay, bisexual and other MSM that has described detrimental consequences of chemsex on mental health [7, 17–20, 33, 34]. MSM respondents in both the UK and US report adverse mental health effects, such as short term depression and paranoia, following chemsex engagement [17–19], and cross-sectional findings have linked chemsex with symptoms of depression and anxiety, although not in multivariate analyses [7, 20]. The most comprehensive assessment of aspects of chemsex users’ psychological wellbeing to date [9], concluded that chemsex users were more likely to report their sexualised drug use having a negative impact on their life. However, an examination of Australian MSM who used GHB explicitly to enhance sexual experiences, often for intensive sex partying, established no correlation between GHB and anxiety or depression [35]. Thus, there is mixed research on the psychosocial impacts of chemsex, the existing studies’ design precludes assessment of the causal direction of the association, and further research with longitudinal designs are warranted. Nonetheless, ours and others’ findings highlight a link between chemsex and MSM’s psychological wellbeing and we encourage consideration of a syndemic perspective, as suggested by e.g. Halkitis and colleagues [2], whereby mental health, drug use, and STI and HIV transmission risks are overlapping and synergistic health challenges, partly driven by the psychosocial vulnerabilities experienced by gay, bisexual and other MSM as sexual minorities.

Taken together, while not all chemsex is problematic in nature, our descriptive findings provide evidence that mirrors previous research on disconcerting health consequences from chemsex. For the large majority of our respondents, chemsex was not a one-time occurrence and research shows that drug use that is dependent or more frequent has more detrimental impacts on people’s psychosocial wellbeing [4]. In concert with other studies on chemsex [4, 9, 16], about half of our respondents reported almost never or never using condoms during chemsex, 10–20% had experienced physical or psychological problems related to chemsex and had engaged in activities during chemsex that they regretted. Thus, these men are at risk of not only accidental overdose, acquisition of STIs, HIV, and Hepatitis, but, as also other researchers have found [3, 9, 14], doing things during chemsex that they would not do when sober. Related, as most research on chemsex has observed, the underlying driver for engaging in chemsex also among our respondents was a type of sex that would be impossible when sober: Great sex that Evan’s [36] informants described as ‘fireworks’. Similar to Pollard and colleagues [11] and informed by a psychodynamic perspective, also Evan [36] concluded that other major drivers of chemsex are loneliness and low self-esteem, again pointing to the need for more research into the mental health aspects of chemsex.

As for implications, healthcare professionals need to be knowledgeable about chemsex and addressing the use of chem-drugs themselves should be part of public health and outreach strategies given their role in the context of mental health and HIV/STI risk behaviors. Chemsex may have a bidirectional link with mental health and our findings suggest that mental health assistance should be among the interventions offered to men engaging in chemsex. Presently, there appears to be no effective biomedical interventions for chemsex [37]. However, in Canada, the vast majority of STI clinic clients indicated being comfortable addressing mental health and substance use concerns with an STI clinic provider [38]. In the UK, drug services tailored to gay, bisexual and other MSM have experienced an exponential rise in attendance [18] and in Australia, there are positive experiences with community-led, harm reduction approaches to chemsex, utilising support services for those seeking to manage or reduce their use, health promotion activities, peer education, and policy work [39]. Thus, such services may provide low barrier and sexual-minority competent care for the two overlapping health challenges. Lastly, given our finding that men who were unemployed had four times greater odds of having reduced mental health, it suggests that unemployment has a negative health impact and indicates the need for social and health policies.

Our findings come with limitations. The current analysis with a cross-sectional study design does not allow for causal inferences. Findings are also limited by possible measurement bias, reliance on self-reports, and selection bias. We sampled mostly well-educated men at only one sexual health clinic in Oslo. The findings may not be generalisable to men not seeking such sexual health services; there are suspicions that STI clinic samples may over-estimate substance use behaviors [4]. Whilst chemsex is more common among men living with HIV, our multivariate analysis did not find this link, which may be because the vast majority of our respondents were HIV-negative. All of that said, the large size of the sample suggests findings are valid, even if some respondents misrepresented self-reports. Other strengths of our study include the use of validated measures, multivariate analyses, and explicit questioning of chemsex. A number of studies measure the use of chem-drugs and sexual behaviors, not the use of specific substances in order to modify sexual sessions.

## Conclusions

Our study contributes new findings to the literature on chemsex. We provide one of the first assessments demonstrating that not only men who have sex with men, but also men who have sex with women engage in chemsex. Chemsex was somewhat more common among MSM than non-MSM and some characteristics of the behavior differed, such as MSM using more GHB/GBL for chemsex. This analysis also enhances our understanding of the relationship between chemsex and mental health, showing that men who engage in chemsex had two times greater odds of reduced mental health, which affected one in five men. From a prevention and treatment perspective, ways must be found to reduce the burden of these overlapping conditions. STI clinics and related sexual health services may present opportune sites for harm reducing, syndemic service integration. In order to build on the evidence base about chemsex and help support the development of prevention- and treatment services, further investigations should seek to enhance our understanding of the precursors of chemsex and what needs are being met with chemsex, and how these are similar and different for MSM and non-MSM. Research is also warranted into the psychosocial impacts of chemsex and ways to keep people safe from the direct and indirect consequences of chemsex.

## Supplementary Information

**Additional file 1: Supplementary file 1** Survey used for the study. Each question/item with answer options are shown.

## Data Availability

The datasets used and/or analysed during the current study are available from the corresponding author on reasonable request.

## Abbreviations

GBL: Gamma-butyrolactone
GHB: Gammahydroksybutyrate
HIV: Human immunodeficiency virus
HSCL-10: Hopkins Symptom Check List
MDMA: Methylenedioxymethamphetamine
MSM: Men who have sex with men
OR: Odds Ratio
STI: Sexually Transmitted Infection
