# Supplementary material for: Links between chemsex and reduced mental health among Norwegian MSM and other men: results from a cross-sectional clinic survey
Source: BMC Public Health. 2020 Nov 25;20:1785. doi: 10.1186/s12889-020-09916-7 (PMC7690186; doi:10.1186/s12889-020-09916-7)
Supplement: Supplementary file 1 — Additional file 1: Supplementary file 1 Survey used for the study. Each question/item with answer options are shown. [file 12889_2020_9916_MOESM1_ESM.docx]

**Survey for men at Olafiaklinikken**

We are examining recreational drug use, especially party drugs, used during sex (“chem-sex” or “party and play”) and the psychological health of our male patients age 16 and over, who have had sex in the last 12 months.

This survey is 100% anonymous. It is not possible to identify you or connect this questionnaire to your medical journal. The information collected will be used to better our understanding of chem-sex, thereby improving our service here at Olafiaklinikken. It may also be used in lectures to health providers or publication in a medical journal.


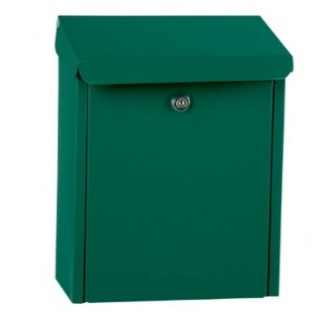


The questionnaire will take 5-10 minutes to complete. When you are finished, please place the form in the secure green mailbox (located on the wall next to the TV screen in the waiting room). Please place the questionnaire in the mailbox, even if you do not manage to complete it fully. If you do not wish to take part, please write the reason here _……………………………………………………………………………………………………………………………..._

Thank you very much for your participation! If you wish to talk to someone about topics addressed in this questionnaire, please mention it to the nurse you meet today.

Many thanks and best wishes from Dr Åse Haugstvedt, consultant physician, Olafiaklinikken.

*NB: If you have completed this questionnaire during a previous visit, we thank you for your participation and ask that you do not complete it again.*

| **Background** |
| --- |

**1.** What is your age? ________ years old

**2.** How many years have you lived in Norway? ______years

**3.** Where do you live? □ Oslo/Akershus □ Elsewhere in Norway □ In another country: ……………………

**4.** Did you come to Norway seeking asylum or as a refugee? □ Yes □ No

**5.** What is your highest education qualification, either completed or started?

□ Primary school □ College or university degree

□ High school/secondary school □ Master’s or Ph.D.

**6.** Which of the following best describes your current situation?

□ Work fulltime or part time □ Student □ Unemployed □ Retired / On disability / Other

**7.** Are you currently in a steady relationship? (In this survey we define a “steady relationship” as having a partner or spouse that means you are not single)

□ Yes, with a man □ Yes, with a woman □ No, I am single □ Other:……………

**8.** Which of the following alternatives best describes you?

□ Bisexual □ Heterosexual □ Gay/homosexual □ Other:………………………..

**9.** Who do you have sex with? □ Women □ Both women and men □ Men

**10.** Are you born a woman but define yourself as a man? (trans) □ Yes □ No

| **Sexual health information** |
| --- |

**11.** In the past 12 months, how many times have you been diagnosed with any of the following sexually transmitted infections / STIs (enter the number of times you have been diagnosed with each STI)

___LGV ___Gonorrhea ___Syphilis ___Chlamydia ___Other

**12.** Have you ever been diagnosed with hepatitis C? □ Yes □ No □ Don’t know

**13.** When was your most recent HIV test?

□ Never tested □ More than 12 months ago □ 3-12 months ago □ Less than 3 months ago

**14.** What was the result of your most recent HIV test?

□ Never tested □ Hiv-positive □ Hiv-negative □ Don’t know

**15.** How often have you used post-exposure prophylaxis /PEP? (taken HIV medicines to reduce the risk of being infected with HIV after possible infection).

□ Never taken PEP □ 1-3 times □ More than 3 times

**16.** PrEP is treatment with HIV medications to reduce the risk of being infected with HIV. Would you like to take PrEP if it became available in Norway?

□ Yes □ No □ Unsure □ Not applicable (I am HIV positive)

| **Sexual behaviors in the last 12 months (place one X for each question)** |
| --- |

**17.** How many different partners have you had sex with in the past 12 months?

□ 1 □ 2-5 □ 6-10 □ 11-20 □ 21-30 □ 31-40 □ 41-50 □ >50

**18.** How often did you use the internet or an app on your phone to find a sexual partner in the last 12 months?

□ Never □ Rarely (<30%) □ Sometimes (30-70%) □ Almost always (>70%) □ Always

**19.** Have you engaged in group-sex the last 12 months? (had sex with 2 or more people at the same time)

□ Yes □ No □ Don’t remember

**20**. Have you participated in an organized sex party the last 12 months?

□ Yes □ No □ Don’t remember

**21.** How often (approximately) did you use condoms when you had vaginal sex with your steady female partner in the last 12 months? (If more than one steady female partner, please tell us about your most recent partner)

□ No steady female partner in the last 12 months □ I have not had vaginal sex

□ Never □ Rarely (<30%) □ Sometimes (30-70%) □ Almost always (>70%) □ Always

**22.** How often (approximately) did you use condoms when you had anal sex with your steady male partner in the last 12 months? (If more than one steady male partner, please tell us about your most recent partner)

□ No steady male partner in the last 12 months □ I have not had anal sex

□ Never □ Rarely (<30%) □ Sometimes (30-70%) □ Almost always (>70%) □ Always

**23.** How often (approximately) did you use condoms when you had vaginal sex with casual female partners in the last 12 months?

□ No casual female partners in the last 12 months □ I have not had vaginal sex

□ Never □ Rarely (<30%) □ Sometimes (30-70%) □ Almost always (>70%) □ Always

**24.** How often (approximately) did you use condoms when you had anal sex with your casual male partners in the last 12 months?

□ No casual male partners in the last 12 months □ I have not had anal sex

□ Never □ Rarely (<30%) □ Sometimes (30-70%) □ Almost always (>70%) □ Always

| **Use of alcohol and drugs** |
| --- |

**25.** When did you last use the following drugs? (Please say when you last did this, even if it was not typical for you.) Place an X for each drug in the relevant time period.

|  | Last week | Last month | Last 6 months | Last 12 months | >12 months ago | Never used |
| --- | --- | --- | --- | --- | --- | --- |
| Alcohol |  |  |  |  |  |  |
| Poppers (amylnitrate) |  |  |  |  |  |  |
| Viagra, Cialis, Levitra or similar (for erection) |  |  |  |  |  |  |
| Cannabis (hash, marijuana) |  |  |  |  |  |  |
| Ecstasy (E, XTC, MDMA) |  |  |  |  |  |  |
| GHB/GLB/ G (liquid ecstacy) |  |  |  |  |  |  |
| Amphetamine (speed) |  |  |  |  |  |  |
| Methamphetamine (crystal, meth, Tina) |  |  |  |  |  |  |
| Heroin or similar drugs (opiates) |  |  |  |  |  |  |
| Mephedrone (4-MMC, meow, methylone) |  |  |  |  |  |  |
| Ketamine (K/Special K) |  |  |  |  |  |  |
| LSD (acid) |  |  |  |  |  |  |
| Cocaine / crack cocaine (crack) |  |  |  |  |  |  |
| Other drugs……………………………………. |  |  |  |  |  |  |

**The next set of questions is about «chem-sex». If you have NOT had «chem-sex» skip to question 39.**

| **Use of recreational drugs during sex («CHEM-SEX») in the last 12 months** |
| --- |

**26.** In the last 12 months, how often did you take recreational drugs during sex? (i.e. engaged in «chem-sex»)

By recreational drugs we mean primarily methamphetamine, GHB/GBL/G, mephedrone, cocaine, ketamine

□ 1 □ 2-5 □ 6-10 □ 11-20 □ 21-30 □ 31-40 □ 41-50 □ >50

**27.** What type of drugs did you use during sex («chem-sex»)? (mark X for any drugs you used)

□ Methamphetamine □ GHB/GBL/G □ Mephedrone □ Cocaine □ Ketamine □ Other:..…………….

**28.** Have you injected drugs (“slamming”) for chem-sex? □ No □ Yes. Which?…………………………..

**29.** If ‘yes’ to question 28, did you share needles with others? □ Never □ Sometimes (30-70%) □ Often (>70%)

**30.** Why do/did you engage in chem-sex? (more than one answer possible)

□ Improve sexual performance □ Enhance sexual experience □ Increase excitement

□ Pressure from sex partners □ Low self-esteem □ Other: ………………………………….

**31.** How do/did you find your chem-sex partners? (more than one answer possible)

□ Cruising location □ Sauna □ Internet □ Darkroom/Sex-club □ Other:…………….

**32.** Where do/did you engage in chem-sex? (more than one answer possible)

□ Cruising location □ Sauna □ Darkroom/Sex-club

□ Sex-party in private home □ Private home □ Hotel □ Other place:………

**33.** In the last 12 months, were you hospitalized for medical issues caused by chem-sex?

□ Never □ 1-3 times □ More than 3 times

**34.** In the last 12 months, were you hospitalized for psychological issues caused by chem-sex?

□ Never □ 1-3 times □ More than 3 times

**35.** Do you wish to stop engaging in chem-sex? □ Yes □ No □ Unsure □ I stopped in the last 12 months

| 36. In the last 12 months how often have you experienced… (mark one X per question) | Never | Rarely (<30%) | Sometimes (30-70%) | Often (>70%) | Always |
| --- | --- | --- | --- | --- | --- |
| - chem-sex as a *problem*? |  |  |  |  |  |
| - *physical* problems in connection with chem-sex? |  |  |  |  |  |
| - *psychological distress* in connection with chem-sex? |  |  |  |  |  |

**37.** How often (approximately) did you or your partners use condoms during vaginal sex or anal sex when you engaged in chem-sex?

□ Never □ Rarely (<30%) □ Sometimes (30-70%) □ Almost always (>70%) □ Always

**38.** Have you participated in a sexual activity during chem-sex that you later regretted or wish you had not participated in? □ No □ Unsure □ Yes. Specify:…………………………………………………………….

| **Mental health** |
| --- |

**39.** In the course of the past 2 weeks, have you been troubled by feeling….? (Please circle the number that best describes your situation)

|  | No | Slightly | Much | Very much |
| --- | --- | --- | --- | --- |
| Sudden fear for no reason | 1 | 2 | 3 | 4 |
| Afraid or anxious | 1 | 2 | 3 | 4 |
| Faint or dizzy | 1 | 2 | 3 | 4 |
| Tense or harassed | 1 | 2 | 3 | 4 |
| Guilty | 1 | 2 | 3 | 4 |
| Sleeplessness | 1 | 2 | 3 | 4 |
| Dejected | 1 | 2 | 3 | 4 |
| Useless, of little worth | 1 | 2 | 3 | 4 |
| That everything is a burden | 1 | 2 | 3 | 4 |
| Hopelessness for the future | 1 | 2 | 3 | 4 |

**40.** Have you ever been diagnosed with ADHD? □ Yes □ No

**41.** In the last 12 months, have you been admitted to a psychiatric ward? □ Yes □ No

**42.** In the last 12 months, did you seriously consider suicide? □ Yes □ No

**43.** In the last 12 months, did you attempt suicide? □ Yes □ No

**44.** At the present time, are you receiving therapy from a psychologist/ psychiatrist/ therapist □ Yes □ No

| **If you ONLY have sex with women, you should NOT answer any more questions.**  **Please place the questionnaire in the green mailbox in the waiting room.**  **Thank you for your help!** |
| --- |

| **QUESTIONS FOR MEN WHO HAVE SEX WITH MEN and men who have sex with men and women** |
| --- |

**45.** Thinking about all the people who know you (family, friends, colleagues, fellow students), what proportion know that you are attracted to men? □ All or almost all □ More than half □ Less than half □ Few □ None

**46**. If you consider yourself bisexual, what was it like "coming out" as bisexual?

□ Easy □ Neither easy nor difficult □ Difficult □ Have not «come out» yet

**47.** If you consider yourself gay, what was it like "coming out" as gay?

□ Easy □ Neither easy nor difficult □ Difficult □ Have not «come out» yet

**48.** To what extent do you consider being gay or bisexual as something negative in and of itself? (Regardless of other people’s possibly prejudices)

□ Not negative at all □ Somewhat negative □ Very negative □ No opinion / Unsure

**Thank you for your help! Please place the form in the green mailbox in the waiting room :-)**
